# Quantification of Drug Transport Function across the Multiple Resistance-Associated Protein 2 (Mrp2) in Rat Livers

**DOI:** 10.3390/ijms16010135

**Published:** 2014-12-24

**Authors:** Pierre Bonnaventure, Catherine M. Pastor

**Affiliations:** 1 Département d’imagerie et des sciences de l’information médicale, Hôpitaux Universitaires de Genève, Geneva 1205, Switzerland; E-Mail: pierre.bonnaventure@unige.ch; 2 Laboratory of Imaging Biomarkers, UMR1149 INSERM-Université Paris Diderot, Sorbonne Paris Cité, Paris 70519, France

**Keywords:** drug hepatic pharmacokinetics, bile excretion rates, drug hepatocyte concentrations, drug bile concentrations, isolated and perfused rat liver

## Abstract

To understand the transport function of drugs across the canalicular membrane of hepatocytes, it would be important to measure concentrations in hepatocytes and bile. However, these concentration gradients are rarely provided. The aim of the study is then to measure these concentrations and define parameters to quantify the canalicular transport of drugs through the multiple resistance associated-protein 2 (Mrp2) in entire rat livers. Besides drug bile excretion rates, we measured additional parameters to better define transport function across Mrp2: (1) Concentration gradients between hepatocyte and bile concentrations over time; and (2) a unique parameter (canalicular concentration ratio) that represents the slope of the non-linear regression curve between hepatocyte and bile concentrations. This information was obtained in isolated rat livers perfused with gadobenate dimeglumine (BOPTA) and mebrofenin (MEB), two hepatobiliary drugs used in clinical liver imaging. Interestingly, despite different transport characteristics including excretion rates into bile and hepatocyte clearance into bile, BOPTA and MEB have a similar canalicular concentration ratio. In contrast, the ratio was null when BOPTA was not excreted in bile in hepatocytes lacking Mrp2. The canalicular concentration ratio is more informative than bile excretion rates because it is independent of time, bile flows, and concentrations perfused in portal veins. It would be interesting to apply such information in human liver imaging where hepatobiliary compounds are increasingly investigated.

## 1. Introduction

Bile excretion is an important function of the liver. Bile forms at canalicular (or apical) membrane of adjacent hepatocytes, which transport endogenous (bile salts and bilirubin) as well as exogenous compounds including drugs [1,2]. In hepatocytes, most transported drugs cross the canalicular membrane by export pumps. These transporters play a major role in drug liver distribution but this topic is difficult to investigate [3]. In humans, duodenal samples can be collected from nasobiliary tubes while bile samples can be obtained when bile duct drainage is necessary in the post-operative period. Being invasive, these approaches are not used routinely. Sandwich-cultured hepatocytes from preclinical species and humans have been used to assess biliary clearance [3,4]. However, this method does not conserve the normal architecture of the liver. Finally, isolation and perfusion of rodent livers conserve an intact architecture of the tissues while the interference with extra-hepatic organs is avoided.

To understand the transport function of drugs across the canalicular membrane of hepatocytes, it is important to measure both hepatocyte and bile concentrations of drugs. It is well known that efflux proteins need ATP to provide energy for uphill transport (from low to high concentrations). However, the concentration gradients across canalicular membrane are rarely provided because repeated liver biopsies would be necessary for each experiment. Whether these high concentration gradients create osmotic gradients that generate transfer fluids into bile canaliculi via water transporters (aquaporins) for all drugs is unknown. The primary canalicular bile produced around adjacent hepatocytes is then modified along ductules and ducts by absorptive and secretory processes that take place in the cholangiocyte epithelium [2].

During the past years, we have investigated the hepatobiliary transport of the contrast agent gadobenate dimeglumine (BOPTA, MultiHance^®^; Bracco Imaging, Milan, Italy) in isolated and perfused rat livers. The drug is used in liver magnetic resonance imaging (MRI) to detect and characterize focal lesions [5,6]. In rats, we showed that BOPTA is transported into bile via the multiple drug resistance associated-protein 2 (Mrp2), no contrast agent being measured in bile when hepatocytes lack Mrp2 [7]. For these studies, BOPTA was labelled with ^153^Gd. We were able to measure liver concentrations by placing a gamma probe that detects ^153^Gd-BOPTA over time (each 20 s) in a region-of-interrest (ROI) of a liver lobe. We also measured liver BOPTA concentrations in rat lacking Mrp2. For comparison, we investigated another hepatobiliary radiotracer used in liver Single Photon Emission Computer Tomography (SPECT) imaging, mebrofenin (MEB, Bridatec^®^; GE Healthcare, Chalfont St. Giles, UK) [8]. MEB cross hepatocytes by the same membrane transporters.

Most experimental studies use bile excretion rates to report drug excretion function in rodent livers. Besides the bile excretion rates of BOPTA and MEB, we investigated several parameters to better define transport function across Mrp2 in isolated and perfused rat liver: (1) Concentration gradients between hepatocyte and bile concentrations over time; and (2) a unique parameter that we named canalicular concentration ratio that represents the slope of the non-linear regression curve between hepatocyte and bile concentrations.

## 2.Results

### 2.1. Bile Flows during Drug Perfusion

**Figure 1:**
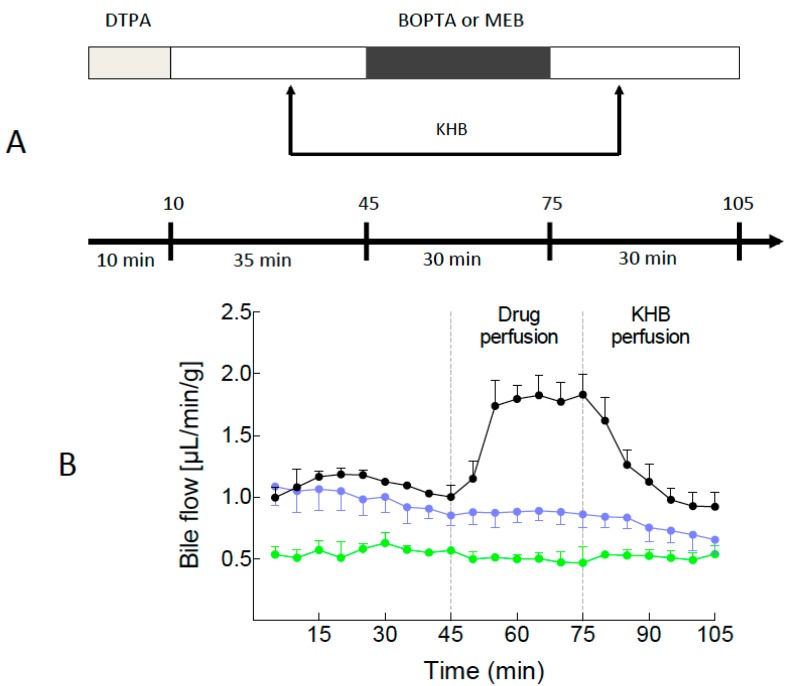
(**A**) Diethylenetriaminepentaacetic acid (DTPA), gadobenate dimeglumine (BOPTA), and mebrofenin (MEB) solutions perfused over time. DTPA is an extracellular contrast agent used in liver imaging that distributes into sinusoids and intersititum while BOPTA and MEB also enter into hepatocytes following extracellular distribution. DTPA was labelled either with ^153^GdCl_3_ or ^99m^Tc while BOPTA was labelled with ^153^GdCl_3_ and MEB with ^99m^Tc. ^153^Gd-DTPA and ^153^Gd-BOPTA were diluted in Krebs-henseleit bicarbonate (KHB) solution to obtain a 200-µM concentration. ^99m^Tc-DTPA and ^99m^Tc-MEB were diluted in KHB solution to obtain a 64-µM concentration. Three groups of rat livers were perfused: (1) Livers isolated from normal rats and perfused with 200 µM DTPA and 200 µM BOPTA (BOPTA group, black circles, *n* = 5); (2) livers isolated from rats lacking Mrp2 and perfused with 200 µM DTPA and 200 µM BOPTA (BOPTA-TR group, green circles, *n* = 3); and (3) livers isolated from normal rats and perfused with 64 µM DTPA and 64 µM MEB (blue circles, *n* = 5); and (**B**) Bile flow rates (µL/min/g of liver) in the three experimental groups over time (min).

Solutions and drugs perfused in the livers over time are illustrated in Figure 1A. Before 45 min, bile flow rates remained steady in the three groups. However, bile flow rates were significantly decreased in the group of livers lacking Mrp2 (Figure 1B). During drug perfusion, bile flow rates significantly increased in BOPTA group (*p* < 0.0001) but remained unchanged in the two other groups. During the rinse period (Krebs-henseleit bicarbonate (KHB) perfusion), bile flow rates returned to baseline values in the BOPTA group while bile flow rates slightly decreased at the end of the protocol in the MEB group.

### 2.2. Canalicular Transport of BOPTA and MEB

**Figure 2:**
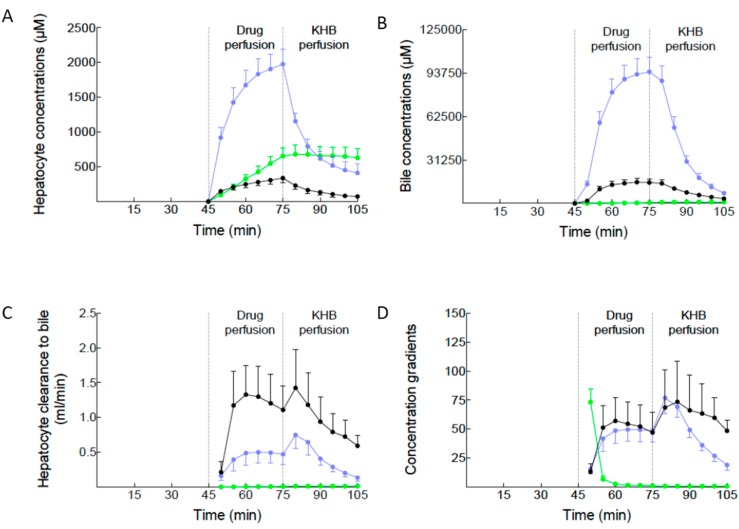
Drug hepatocyte concentrations (**A**); bile concentrations (**B**); hepatocyte clearance into bile (**C**); and concentration gradients between bile and hepatocytes (**D**) over time (min). The three experimental groups are described in Figure 1. During drug perfusion, constant concentrations are perfused. During KHB perfusion, no drug is perfused (rinse period). BOPTA (black circles, *n* = 5); BOPTA in Mrp2-deficient livers (green circles, *n* = 3); MEB (blue circles, *n* = 5). Hepatocyte concentrations were available every 20 s, but presented in the graph every 5 min.

To assess the canalicular transport of BOPTA and MEB across Mrp2, we calculated the concentration gradients across the membrane. Hepatocyte concentrations increased steadily during the drug perfusion period for the two drugs (Figure 2A). BOPTA concentrations were significantly higher in the absence than in the presence of Mrp2. MEB concentrations were much higher than those of BOPTA. During the rinse period, all concentrations decreased except those measured in the BOPTA-TR (no Mrp2) group. Thus, in the absence of Mrp2, BOPTA remained trapped in hepatocytes.

Tiny BOPTA bile concentrations were measured in this group (Figure 2B). BOPTA and MEB bile concentrations greatly increased during the drug perfusion period until 150,269 ± 2764 µM (BOPTA) and 94,703 ± 10,703 µM (MEB). During the rinse period, drug bile concentrations progressively decreased in normal livers.

Hepatocyte clearances of drugs into bile (mL/min) significantly differed in the three experimental groups (Figure 2C). The maximal hepatocyte clearances into bile occurred 5 min after the stop of drug perfusion when livers were perfused with KHB solution. At this time-point, BOPTA had higher clearances (1.4 ± 0.6 mL/min or g of hepatocytes/min) than MEB (0.8 ± 0.2 mL/min or g of hepatocytes/min).

The gradients between drug bile and hepatocyte concentrations estimate Mrp2 function over time (Figure 2D). These gradients are not steady over time: they increased at the beginning of the perfusion period and reached a plateau. During this period, gradients were similar for BOPTA and MEB (close to 50). The gradients increased at the beginning of the rinse period with both drugs before a slight decrease for BOPTA and a more pronounced decrease for MEB. Gradient of BOPTA in the absence of Mrp2 rapidly dropped to 0.

**Figure 3:**
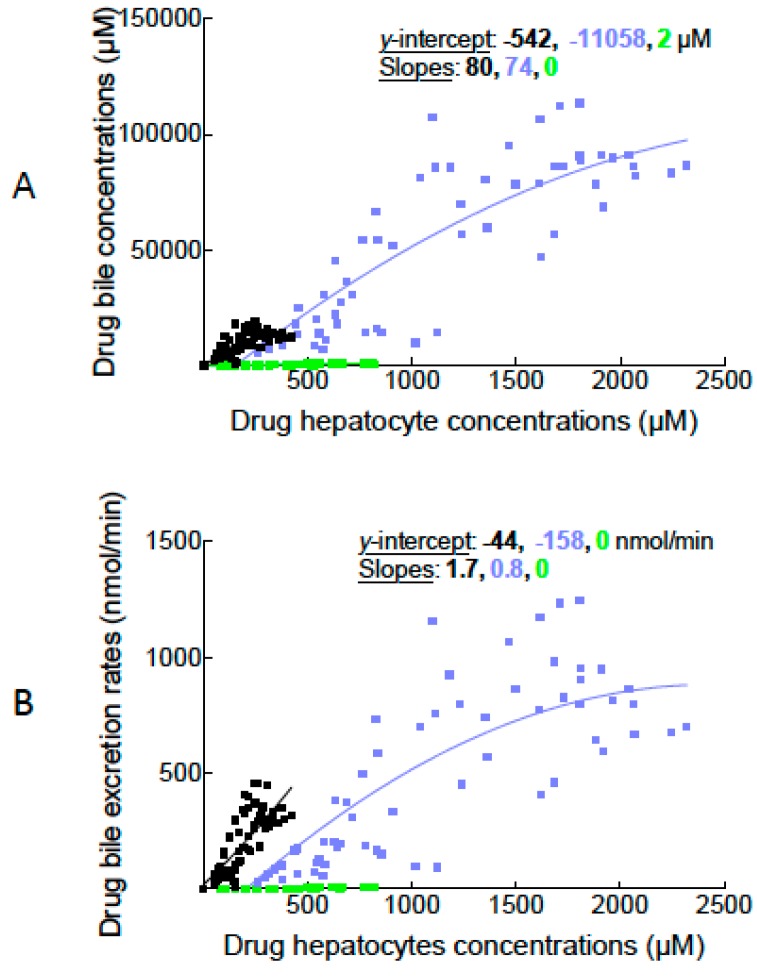
Plots between drug hepatocyte concentrations (*x*-axis) and drug bile concentrations (*y*-axis) (**A**) or drug bile excretion rates (*y*-axis) (**B**). The three experimental groups are described in Figure 1. BOPTA (black squares, *n* = 5); BOPTA in Mrp2-deficient livers (green squares, *n* = 3); MEB (blue squares, *n* = 5).

Another way to illustrate MEB and BOPTA canalicular transport through Mrp2 is to plot drug hepatocyte concentrations (*x*-axis) and drug bile concentrations (*y*-axis) when available (Figure 3A). To analyse non-linear regression curves, we fitted experimental values with the equation A + B*X* + C*X^2^*, where A is intercept, B is slope, and C is plateau at maximal concentrations. All curves were significantly different (*p* < 0.0001). However, when only B (slope of the regressions) was compared in BOPTA (80) and MEB (74) groups, values were similar (*p* = 0.90). The shape of the non-linear regressions also confirm that the transport of drugs through Mrp2 is saturated for high concentrations.

We also plotted drug hepatocyte concentrations (*x*-axis) and drug bile excretion rates (*y*-axis) (Figure 3B). Curves in BOPTA and MEB groups were significantly different (*p* < 0.0001) when the three parameters A, B, and C were compared together. When only B was compared between BOPTA (0.7) and MEB (1.4) groups, le parameter was similar (*p* = 0.20).

**Figure 4:**
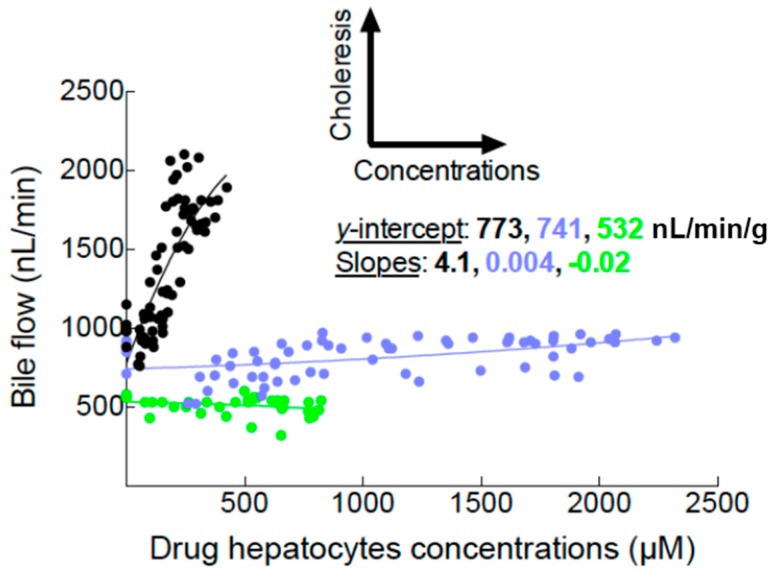
Plots between drug bile excretion rates (*x*-axis) and bile flow rates (*y*-axis). The three experimental groups are described in Figure 1. BOPTA (black circles, *n* = 5); BOPTA in Mrp2-deficient livers (green circles, *n* = 3); MEB (blue circles, *n* = 5).

Finally (Figure 4), to understand how canalicular fluid transport, bile concentrations, or both interfere with drug excretion rates into bile, we plotted drug bile excretion rates (*x*-axis) with bile flow rates (*y*-axis). The relation clearly shows that bile excretion rates of MEB are driven by bile concentrations while in the BOPTA group, drug bile excretion rates increased according to bile concentrations and choleresis.

## 3. Discussion

### 3.1. Assessment of Mrp2 Function in the Entire Rat Liver

Most experimental studies use bile excretion rates to report drug excretion function in rodent livers. Besides the bile excretion rates of BOPTA and MEB, we measured in our experimental model several parameters to better define transport function across Mrp2: (1) Concentration gradients between hepatocyte and bile concentrations over time; and (2) a unique parameter that we named canalicular concentration ratio that represents the slope of the non-linear regression curve between hepatocyte and bile concentrations. Interestingly, despite different transport characteristics including bile excretion rates, BOPTA and MEB have a similar canalicular concentration ratio. In contrast, the ratio was null in hepatocytes lacking Mrp2.

The canalicular concentration ratio we described is more informative than bile excretion rate for several reasons. One obvious reason is that modifications of transfer fluid across membrane related to drug structure change the bile excretion rates. BOPTA is a choleretic compound while the transport of MEB does not change bile flow rates. Thus, the bile excretion rates of BOPTA depend on drug concentrations and fluid transfer while the bile excretion rates of MEB depend only on drug concentrations. Tavoloni *et al.* [9] previously reported the relationship between bile excretion rates and bile flow as shown in Figure 4 to differentiate the effects of fluid transfer and bile drug concentrations. Another reason is that bile excretion rates are conditioned by hepatocyte uptake of drugs. Then, bile excretion rates reflect both the uptake and bile excretion of drugs. The only way to assess Mrp2 function independently from cell uptake is to measure the concentration gradients between hepatocytes and bile. In our experimental protocol, the gradients highly differ over time. However, the concentrations gradients measured for MEB and BOPTA were similar over time except at the end of the protocol.

The gradients greatly change over time and cannot quantify the Mrp2 function by a single parameter. To assess Mrp2 function, we then use the unique parameter given by the slope of the non-linear regressions between hepatocyte and bile concentrations. The parameter was similar for BOPTA and MEB in normal livers. This parameter is important because it is independent of the time of bile sampling, drug concentrations perfused in portal vein, and drug-induced choleresis or cholestasis. Finally, the canalicular concentration ratio was null in rat livers deficient in Mrp2. In future studies, we need to measure the ratio in the presence of Mrp2 inhibitors. In such case, we might be able to quantify the inhibition of Mrp2 function independently from a putative simultaneous inhibition on hepatocyte uptake function. Moreover, the canalicular concentration ratio and the equation *Y* = A + B*X* + C*X*^2^ are not widely used in pharmacological research and need additional validations in various experimental conditions. Thus, the intercept (A), slope (B), and C (plateau) of the relation would be better defined.

### 3.2. Drug Hepatic Pharmacokinetics in Isolated Perfused Rodent Livers

Isolated and perfused rodent livers are important experimental model to investigate the hepatic pharmacokinetics of drugs [10]. Various types of data can be measured in this model. The main advantages are the well-controlled and simplified conditions of experiments. Isolation of the liver eliminate the pharmacokinetic interferences that originate from extra-hepatic organs. Solutions do not contain proteins, all molecules being free to enter into hepatocytes. Livers are perfused only through the portal vein and it is possible to perfuse constant concentrations of drugs. Thus, we can compare for BOPTA the inflow concentration (200 µM), the maximal hepatocyte concentrations (338 ± 67 µM), and the maximal bile concentrations (15,012 ± 2399 µM). MEB generates higher gradients between the various compartments: 64 µM in portal vein, 1977 ± 117 µM in hepatocytes, and 94,703 ± 10,709 µM in bile.

### 3.3. Calculation of Hepatocyte Concentrations

In studies that focus on drug hepatic pharmacokinetics, the concentration gradients across the canalicular are rarely reported. When these gradients are reported, only a few time-points were assessed, successive liver biopsies being difficult to obtain over time [11,12]. The originality of our model is that drugs are labeled with ^153^Gd or ^99m^Tc, and liver concentrations are measured over time in a ROI by a gamma probe placed over rat livers. Because BOPTA is a MRI contrast agent, we also isolated and perfused rat liver in the MRI room to assess the hepatic pharmacokinetics of the drug by measuring hepatic signal intensities [13,14]. To our knowledge, the radiotracer MEB has never been perfused in isolated rat livers with a µ-SPECT device. In liver ROI, various compartments are present such as sinusoids, interstitium, hepatocytes, other cells such as macrophages, and bile canaliculi. Drug concentrations in each compartment of the ROI delineated by the probe, must be taken into consideration. This is the reason why we substracted the Diethylenetriaminepentaacetic acid (DTPA) concentrations of sinusoids and interstitium from the liver concentrations of BOPTA and MEB. Besides hepatocytes, the other compartment that can contain hepatobiliary drugs are bile canaliculi and we also withdrew bile canaliculi concentrations. Blouin *et al.* [15] previously measured this volume at 0.43%. We are not aware of another method to measure concentrations in such compartment. Although the volume is tiny, the maximal BOPTA concentrations were 62 ± 11 µM for BOPTA and 383 ± 76 µM for MEB. Moreover, we must assume that drug concentrations in bile canaliculi and bile duct are similar. However, the composition of primary bile formed inside canaliculi is modified by solutes and water transported across cholangiocytes along bile ductules and ducts. According to a recent review [2], the modification of primary bile along ductules should be less than 10% in rats. Because the count rates of radioactivity measured by the probe originate from 78% of ROI (hepatocyte volume), we should increase the calculated values by 22% to obtain the true concentrations in hepatocytes. In the figures, this correction is not done.

### 3.4. Transport Function of Rat Mrp2

Mrp2 is an organic anion transporter expressed in the apical membrane of polarized cells such as hepatocytes, renal cells, and enterocytes [3]. In hepatocytes, Mrp2 excretes glucuronide and sulfate conjugates of bile acids. Mrp2 is also responsible for glutathione biliary transport. Glutathione is a major osmolyte partly responsible for bile-salt-independent bile flow. Mrp2 generates a steep concentration gradient of glutathione from 100 µM in blood to 10 mM in bile. As shown in our study, in Mrp2-deficient rats, bile flow rate is lower (−50%) than in normal rodents [3]. Similarly to glutathione, we measure a high BOPTA concentration gradient from 15,000 µM in bile to 200 µM in portal vein.

Besides BOPTA, MEB is avidly taken up by human [16,17] and rat livers [18], as well as isolated hepatocytes [19]. We find a high accumulation of MEB in rat hepatocytes. The maximal bile excretion rate was much higher than that of BOPTA. MEB has no choleretic or cholestatic effect. Previous publications showed that MEB in human hepatocytes is taken up by OATP1B1 (Organic Anion-Transporting Peptide B1) and OATP1B3, but not by Na^+^-Taurocholate Cotransporting Polypeptide (NTCP) and OATP2B1 [20,21]. In rats, MEB hepatic uptake is predominantly Oatp-mediated and the tracer is preferentially excreted unchanged into bile by Mrp2 [22,23,24].

## 4. Experimental Section

### 4.1. Experimental Groups

We extracted and re-analysed data from two experimental groups that were previously published [7]. In this article, the liver concentrations of BOPTA were measured but hepatocyte and bile canalicular concentrations were not extracted. The two groups of rat livers were perfused with the same protocol: (1) Livers isolated from normal rats and perfused with 200 µM gadobenate dimeglumine (BOPTA, MultiHance^®^, Bracco Diagnostics, Milan, Italy) (BOPTA group, *n* = 5); and (2) livers isolated from rats deficient in Mrp2 and perfused with 200 µM BOPTA (BOPTA-TR group, *n* = 3). A third group of rat livers was added. In this group, 64 µM mebrofenin (MEB, Bridatec^®^, GE Healthcare, Chalfont St. Giles, UK) was perfused (MEB group, *n* = 5). Male Sprague-Dawley rats (Charles River, Les Arbreles, France) were anesthetized with pentobarbital (50 mg/kg, intraperitoneal injection). All animals received humane care according to the criteria outlined by the veterinary office in Geneva (Switzerland), which approved the protocol.

### 4.2. Isolation and Perfusion of Rat Livers

Livers were isolated leaving the organ in the carcass. The abdominal cavity was opened and the portal vein was cannulated and secured. A G_16_ catheter was introduced into the portal vein up to 2–3 mm from the liver. After portal vein cannulation, the abdominal vena cava was transected and a Krebs-Henseleit-bicarbonate (KHB) solution (118 mM NaCl, 1.2 mM MgSO_4_, 1.2 mM KH_2_PO_4_, 4.7 mM KCl, 26 mM NaHCO_3_, 2.5 mM CaCl_2_) was pumped without delay into the portal vein, the solution being discarded by the transection. The flow rate was slowly increased over one minute up to the desired value (30 mL/min) to avoid sinusoid injury potentially induced by the rapid increase of sinusoidal pressures. In a second step, the chest was opened and a second cannula inserted through the right atrium. This catheter collect solutions leaving the liver through hepatic veins. Finally, abdominal inferior vena cava was ligatured allowing solutions perfused by the portal vein to be eliminated by hepatic veins. The entire perfusion system consisted of reservoir, pump, heating circulator, bubble trap, filter, and oxygenator. The solution of perfusion was equilibrated with a mixture of 95% O_2_:5% CO_2_. The livers were perfused with a KHB buffer ± drugs using a non-recirculating system, livers being always perfused by fresh solutions. In each experiment, the common bile duct was cannulated with a PE_10_ catheter and bile samples collected every 5 min. Samples of hepatic veins were also collected each 5 min. Livers were perfused with a constant liver flow rate: 30 mL/min.

### 4.3. Drugs and Experimental Protocol (Figure 1A)

Several drugs were perfused: gadopentetate dimeglumine (DTPA, Magnevist^®^, Bayer Pharma, Berlin, Germany), BOPTA, and mebrofenin MEB. DTPA distributes into sinusoids and intersititum of rat liver and is labelled with either ^153^GdCl_3_ (0.5 M DTPA solution, 1 MBq/mL) or ^99m^Tc (25 mg, 7 MBq). BOPTA enters into hepatocytes and is excreted into bile and is labelled by adding ^153^GdCl_3_ to a 0.5 M BOPTA solution (1 MBq/mL). MEB was labelled by adding ^99m^Tc to MEB (40 mg, 11 MBq). ^153^Gd-DTPA and ^153^Gd-BOPTA were diluted in KHB solution to obtain a 200-µM concentration and ^99m^Tc-DTPA and ^99m^Tc-MEB were diluted in KHB solution to obtain a 64-µM concentration.

Livers were successively perfused with 200 µM ^153^Gd-DTPA (10 min), KHB solution (35 min), 200 µM ^153^Gd-BOPTA (perfusion period of 30 min), and KHB solution (rinse period, 30 min) or 64 µM ^99m^Tc-DTPA (10 min), KHB solution (35 min), 64 µM ^99m^Tc-MEB (perfusion period of 30 min), and KHB solution (rinse period, 30 min).

### 4.4. Drug Concentrations in Hepatocytes

To quantify drug liver concentrations, we placed a gamma scintillation probe that measures radioactivity inside the liver. Count rates are provided every 20 s. The probe measures the radioactivity in a ROI inside a liver lobe. This ROI is constant over the entire protocol and between experiments. To transform radioactivity counts into contrast agent concentrations, the radioactivity in the liver at the end of each experiment was measured (Activimeter Isomed 2000; Nuklear-Medizintechnik Dresden GmbH, Dresden, Germany) and related to the last count measured by the probe.

To calculate hepatocyte concentrations, we withdraw the liver concentrations measured during the perfusion of DTPA as well as concentrations in bile canaliculi from total liver concentrations of BOPTA or MEB. Indeed, the ROI includes several compartments with various volumes: sinusoids (10%), interstitium (5%), hepatocytes (78%) and bile canaliculi (0.43%) [15]. Drug concentrations in bile canaliculi were calculated by multiplying bile concentrations measured in the main bile duct by the volume of bile canaliculi present in ROI (0.43%). Although the volume is tiny, BOPTA and MEB concentrations in canaliculi are not negligible. DTPA, BOPTA, and MEB concentrations in bile were measured every 5 min with a Packard Cobra auto-gamma counter (Camberra Packard, Switzerland). Concentrations were expressed in µM (corresponding to nmol/mL) in bile duct, as well as in livers, 1 g of liver being close to 1 mL. Bile excretion rates (in nmol/min) were calculated by the following formula: C_BILE_x bile flow rate. Concentrations in bile (C_BILE_) is measured in µM and bile flow in µL/min. Bile flow was expressed in µL/min/g. Hepatocyte clearance into bile was also calculated. Hepatocyte clearance into bile (mL/min or g of hepatocytes/min) = bile excretion rates (nmol/min)/hepatocyte concentrations (nmol/mL or g of hepatocytes).

### 4.5. Statistics

Parameters are means ± S.D. Kruskal-Wallis tests were performed to compare the mean values between experimental groups. To compare the evolution of concentrations over time we use a two-way ANOVA with multisple comparisons between groups within each time-points (Prism 6, GraphPad; GraphPad-Prism Software Inc., San Diego, CA, USA).

To evidence the transport function of Mrp2, we plotted various values (for example hepatocyte *vs.* bile concentrations). We then analysed the relationship with non-linear regressions, which fit the experimental values with the equation A + B*X* + C*X^2^*, A, B, and C representing the intercept on *y*-axis, slope, and C (plateau) respectively (Prism 6, GraphPad). The goodness of fit of each data set was measured by the extra sum-of-squares *F* test. Then, comparisons between data sets were performed to determine whether curves were statistically indistinguishable (*F* test). This test compares concomitantly the A, B, and C parameters of the three data sets. Moreover, with this sofware, it is possible to compare a single parameter (such as B or slope of regression) between different groups.

## 5. Clinical Relevance of the Study

The quantification of Mrp2 function was investigated in isolated and perfused rat livers using BOPTA and MEB, two hepatobiliary compounds in clinical liver imaging. Both techniques detect and characterize focal liver lesions as well as hepatic function assuming that the higher the accumulation of BOPTA or MEB in hepatocytes, the better the function [5,8]. However, as seen with the high accumulation of BOPTA in liver lacking Mrp2, the interpretation of liver imaging is not straightforward. An apparent high accumulation of drugs in the liver might correspond to lack of excretion when drugs were capable of entering hepatocytes. Consequently, understanding the function of Mrp2 independently from drug hepatocyte uptake is valuable. Previous experimental studies used liver imaging to investigate the hepatobiliary transport of tracers [25,26]. The transfer of our results to patients relies on an understanding of species differences for drug transporters [27] and scaling factors that relate *in vitro* transporter data to accurate *in vivo* predictions [24]. These issues being challenging, it should be possible soon to estimate with liver imaging both liver and bile concentrations and to calculate the canalicular concentration ratios in patients [28,29].
